# Longitudinal Assessment of Exercise Capacity via Cardiopulmonary Exercise Testing in Heart Failure Patients with Improved and Persistently Reduced Ejection Fraction: Results from the IMPRO_2_VEMENT-HF Study

**DOI:** 10.3390/jcm15218406

**Published:** 2026-10-28

**Authors:** Diana Ladera-Santos, José López-Aguilera, Jesús Alberto Torres-Zamudio, Consuelo Fernández-Avilés, Jorge Perea-Armijo, Guillermo Gutiérrez Ballesteros, Gloria Heredia Campos, Luis Carlos Maestre Luque, Rafaela Luque López, Asunción García Merino, Ángela Heredia Torres, Juan Manuel García Medina, José María Segura Saint-Gerons, Álvaro Roldán-Guerra, Manuel Crespín-Crespín, Mónica Delgado-Ortega, Rafael González Manzanares, Dolores Mesa-Rubio, Manuel Pan-Álvarez Osorio, Manuel Anguita-Sánchez, Juan Carlos Castillo-Domínguez, Martín Ruiz-Ortiz

**Affiliations:** 1 Cardiology Department, Reina Sofia University Hospital, 14011 Cordoba, Spain; dianaladerasantos2@gmail.com (D.L.-S.); mircardjla@gmail.com (J.L.-A.); alvaroroldan97@gmail.com (Á.R.-G.); loladoctora@gmail.com (D.M.-R.);; 2 Cordoba Biomedical Research Institute, Instituto Maimónides de Investigación Biomédica de Córdoba (IMIBIC), 14011 Cordoba, Spain; 3 Cardiac Rehabilitation Unit, Cardiology Department, Reina Sofia University Hospital, 14011 Cordoba, Spain; 4 Heart Failure Unit, Cardiology Department, Reina Sofia University Hospital, 14011 Cordoba, Spain; 5 Centro de Investigación Biomédica en Red en Enfermedades Cardiovasculares (CIBERCV), Carlos III Institute of Health, 28029 Madrid, Spain; 6 Faculty of Health Sciences, Universidad Internacional Isabel I de Castilla, 09003 Burgos, Spain

**Keywords:** CPET, improved ejection fraction, functional capacity, ventricular remodeling

## Abstract

**Background:** Patients with heart failure and reduced ejection fraction (*HFrEF*) who achieve improved ejection fraction (*HFimpEF*) after guideline-directed medical therapy exhibit superior prognosis. However, functional impairment remains a hallmark of *HF*. **Aims**: To characterize and compare exercise capacity profiles via cardiopulmonary exercise testing (*CPET*) between patients with *HFimpEF* and those with persistently reduced ejection fraction (*HFprEF*) using a baseline-adjusted analysis. **Methods:** Data from a longitudinal cohort of 111 *HFrEF* patients with baseline and follow-up *CPET* data (minimum 6-month follow-up) were analyzed. Patients were stratified into *HFimpEF* and *HFprEF* groups, according to the Universal Definition of *HF*. Changes between groups were evaluated using Analysis of Covariance (ANCOVA) adjusted for baseline values, with non-parametric Bootstrapping (2000 resamples) and multiplicity. **Results:** We included 111 patients: 69 with *HFimpEF* (62.2%) and 42 with *HFprEF* (37.8%). At a median follow-up of 8.7 months, the *HFimpEF* group showed significantly greater reverse remodeling (adjusted ΔLVEF +19.9%, *p* < 0.001) and a greater reduction in *NT-proBNP* levels (−557.6 pg/mL, *p* = 0.042). Both groups demonstrated intra-group functional improvements. However, in adjusted between-group ANCOVA models, *HFimpEF* achieved a significantly greater recovery in percentage of predicted peak oxygen consumption (adjusted difference: +8.5%; 95% CI: 1.2 to 16.1; *p* = 0.021). Net adjusted between-group differences for absolute *VO*_2_*peak* gain, submaximal threshold, ventilatory efficiency and recovery kinetics did not reach statistical significance. Multivariate analysis identified older age (beta −0.261, *p* = 0.010), diabetes mellitus (beta −0.210, *p* = 0.023) and baseline *VO*_2_*peak* (beta −0.569, *p* < 0.001) as negative predictors of improvement. **Conclusions:** Comprehensive *HFrEF* management improves exercise capacity across all patients. Although *HFimpEF* yields superior gains in normalized aerobic capacity, overall functional trajectories are largely comparable between phenotypes. This structural-functional decoupling underscores persistent residual limitations in *HFimpEF*, supporting CPET for longitudinal risk stratification.

## 1. Introduction

*HF* is characterized by a profound reduction in functional capacity, manifesting as severe exercise intolerance that restricts essential activities of daily living due to the onset of dyspnea, fatigue, or anginal symptoms [1]. *CPET* remains the gold standard for the objective quantification of these limitations [1,2,3,4]. In clinical practice, peak oxygen consumption (*VO*_2_*peak*) is the primary metric employed to gauge exercise capacity, as it provides a reliable reflection of systemic metabolic reserves [5]. However, since *HF* patients often cannot achieve maximal effort, submaximal variables have gained prominence as critical indicators. These parameters are essential for characterizing exercise capacity and serve as robust predictors of medium-term prognosis, including all-cause mortality, the necessity for cardiac transplantation, and the risk of *HF*-related readmissions [6,7,8].

Current evidence suggests that approximately 30–40% of patients with *HFrEF* exhibit significant recovery of left ventricular function during follow-up [9]. According to the Universal Definition of *HF* [10], patients with a baseline *LVEF* ≤ 40% who demonstrate an absolute increase of ≥10 points and a second measurement > 40% are classified as *HFimpEF*. Conversely, those failing to meet these criteria are categorized as having *HFprEF*. This *HFimpEF* phenotype is characterized by distinct clinical profiles and a markedly more favorable prognosis. Given that *VO*_2_*peak* is determined by both central hemodynamic and peripheral factors [1,11], it is hypothesized that patients with *HFimpEF* achieve a superior increase in exercise capacity—as quantified by *CPET*—compared with those with *HFprEF* [12]. This enhancement reflects greater metabolic and ventilatory efficiency secondary to left ventricular reverse remodeling. Nevertheless, while their exercise tolerance improves significantly, it often remains below that of healthy cohorts, with residual symptoms persisting in a substantial proportion of patients.

### AIMs

The primary aim of this study is to characterize and compare the exercise capacity, as quantified by *CPET,* between patients with *HFimpEF* and those with *HFprEF* during clinical follow-up. Other secondary objectives are: (1) to analyze predictors of improvement in peak oxygen consumption in follow-up; (2) to evaluate the neurohormonal profile of the *HFimpEF* cohort by analyzing longitudinal variations in NT-proBNP levels; and (3) to assess left ventricular reverse remodeling by comparing echocardiographic parameters between patients with *HFimpEF* and those with *HFprEF*.

## 2. Materials and Methods

### 2.1. Study Design and Population

This work, the *IMPRO*_2_*VEMENT-HF* study, is an observational, retrospective, longitudinal, and analytical study of a cohort of *HFrEF* patients. The study population includes individuals who underwent exercise capacity assessment via *CPET* following either hospitalization or an outpatient consultation for *HF* symptoms. Participants were required to have a baseline *CPET* and a subsequent follow-up assessment conducted after the optimization of *GDMT*. The inclusion period spanned from June 2019 to December 2023.

### 2.2. Cohort Stratification

Patients were stratified into distinct clinical phenotypes based on longitudinal echocardiographic data and the evolution of their LVEF, in accordance with the universal definition of HF [10]: Cohort 1 (*HFimpEF*) and Cohort 2 (*HFprEF*).

### 2.3. Ergoespirometric Variables

Exercise capacity was assessed by means of an incremental ramp CPET on a treadmill, using a Schiller MTM-1500 treadmill coupled with a Ganshorn Powercube^®^ gas analyzer. The protocol was selected according to each patient’s clinical characteristics and estimated functional capacity, with the aim of achieving an estimated exercise duration of 8–12 min. The Naughton protocol and three protocols specifically designed for patients with heart failure—EXTRA SOFT, SOFT, and SOFT-STAND—were used. To ensure comparability of exercise duration between the baseline and follow-up CPET assessments, each patient performed the same protocol ab both evaluations.

The Naughton protocol was performed at a constant speed of 3.2 km/h, with the treadmill grade increased by 3.5% every 2 min, starting at a 0% incline. Each of the other three protocols included a 2 min warm-up period, an incremental exercise phase lasting up to 13 min, and a 2 min recovery period. In the EXTRA SOFT protocol, the speed was initially set at 2 km/h and progressively increased to 8 km/h, while the treadmill grade increased from 4% to 9%. In the SOFT protocol, speed increased from 2 to 9 km/h and treadmill grade from 4% to 12%. Finally, in the SOFT-STAND protocol, speed increased from 2 to 11 km/h and treadmill grade from 10% to 15%.

The test was terminated upon symptom-limited exhaustion or the appearance of clinical, electrocardiographic, or metabolic termination criteria, including an RER ≥ 1.10, which was considered a specific criterion for patients with heart failure.

Aerobic capacity and gas exchange parameters included: Peak oxygen consumption (*VO*_2_*peak*), mL/kg/min and as a percentage of the predicted value (*predicted %VO*_2_*peak*), as well as oxygen consumption at the first ventilatory threshold (*VO*_2_-*VT1*) and the time required to reach VT1 (*t-VT1*). *VT1* was determined using the oxygen equivalent method (*VE*/*VCO*_2_) and the V-slope method from the Wasserman Panel 5 (the point where the linearity of the VCO_2_ vs. VO_2_ slope breaks) [13,14]. When the results from these two methods did not align exactly, the oxygen equivalent method was given greater weight. Furthermore, maximal exercise time during *CPET*, measured in minutes, was also considered. Based on the *predicted %VO*_2_*peak* achieved by the patient during *CPET*, functional capacity was classified as: (1) good (VO_2_ > 110%), (2) normal (VO_2_ > 90%), (3) lower limit of normal (VO_2_ 80–90%), (4) mildly reduced (VO_2_ 60–80%), (5) moderately reduced (VO_2_ 50–60%), and (6) severely reduced (VO_2_ < 50%).

Ventilatory Efficiency was evaluated via the *VE/VCO*_2_ *slope* (and the Weber functional classification (I–IV)), the Oxygen Uptake Efficiency Slope (*OUES%*). Additionally, end-tidal gas pressures (*PETO*_2_ and *PETCO*_2_) and equivalent fractions (*VE/VCO*_2_ and *VE/VO*_2_) were recorded at baseline, VT1, and peak exercise.

Hemodynamic and chronotropic responses were characterized by heart rate (HR) at rest, at VT1, and at peak exercise, and oxygen pulse (mL/beat and %predicted).

Recovery kinetics were assessed through the RER recovery ratio (*RER-2minRec*), and the *Recovery Delay*, measured in seconds. Finally, the *MECKI Score* (%) was calculated as a composite prognostic index.

### 2.4. Clinical, Analytical, Electrocardiographic, Echocardiographic and Treatment Variables

Secondary variables encompassed a comprehensive suite of clinical, biochemical, electrocardiographic, and echocardiographic parameters. Pharmacological optimization was monitored by recording the prescription and titration of neurohormonal antagonists at baseline and follow-up, specifically including betablockers, angiotensin-converting enzyme inhibitors (*ACEi*), angiotensin II receptor blockers (*ARBs*), angiotensin receptor-neprilysin inhibitors (*ARNIs*), mineralocorticoid receptor antagonists (*MRAs*), and sodium-glucose cotransporter-2 inhibitors (*SGLT2i*), alongside diuretic therapy. Furthermore, the presence of cardiac electronic devices was documented, including implantable cardioverter-defibrillators (*ICDs*) and cardiac resynchronization therapy, which in our cohort was exclusively performed via Left Bundle Branch Area Pacing (*CRT-LBBAP*) in all cases.

The Cardiac Rehabilitation Program (*CRP*) for patients with *HF* was developed in a structured and individualized manner, with exercise prescription tailored to each patient’s clinical status, functional capacity, and individual characteristics. The training program combined aerobic and resistance exercise. Aerobic exercise was generally performed two to three days per week, in sessions lasting approximately 60 min, using modalities such as walking, cycling, or treadmill exercise. The initial intensity was moderate and was progressively increased according to the patient’s tolerance. Resistance training was performed in combination during the same sessions, one to two times per week, using exercises targeting the major muscle groups and elastic bands, resistance machines, free weights, or body-weight exercises. Typically, participants performed one to three sets of 8–16 repetitions per exercise. The program was complemented by health education, promotion of heart-healthy lifestyle habits, and strategies aimed at improving adherence to treatment and exercise. Treatment adherence was strictly defined as attendance at ≥75% of the prescribed *CRP* sessions during the 3-month duration of the complete cardiac rehabilitation program.

### 2.5. Ethical Aspects

The study was approved by the Córdoba Research Ethics Committee under approval number TFG-FERE-2021. Given the retrospective design and the use of anonymized data, the Ethics Committee granted a waiver of the requirement for informed consent. Data were handled anonymously and in accordance with applicable data protection regulations. The data supporting the findings of this study are available from the corresponding author upon reasonable request, while respecting participant confidentiality. The study was conducted in accordance with the Declaration of Helsinki and applicable national ethical regulations.

### 2.6. Statistical Analysis

Data normality was assessed using the Kolmogorov–Smirnov test for samples exceeding 50 cases and the Shapiro–Wilk test for smaller cohorts. Continuous variables are presented as mean ± standard deviation (SD) for normally distributed data or as median and interquartile range (IQR) for non-normally distributed variables. Categorical data are reported as absolute frequencies and percentages (n, %).

For univariate comparisons of continuous variables, the *t*-test for paired or independent samples was employed where appropriate. For non-parametric distributions, the Wilcoxon signed-rank test or the Mann–Whitney U test was utilized. To directly compare the magnitude of change between clinical evolution groups (*HFimpEF* vs. *HFprEF*) while accounting for violations of normality and variance homogeneity, a robust analysis was performed. Specifically, an Analysis of Covariance (ANCOVA) was conducted on the follow-up values of continuous variables, utilizing their respective baseline values as covariates. To ensure the validity of the statistical inferences under heteroscedasticity and non-normal distributions, the ANCOVA model was coupled with a non-parametric Bootstrapping approach based on 2000 resamples. Statistical significance and effect sizes were determined using bias-corrected and accelerated (BCa) 95% confidence intervals (95% CI) and partial eta squared values, respectively. As a sensitivity analysis, to compare the change in ergoespirometric variables between groups, new variables were calculated as the difference between follow-up and baseline values. These differences were compared with the previously mentioned tests for independent variables, as appropriate. In case of discrepancy, the significance values obtained by the ANCOVA method were preferred.

Categorical variables were analyzed using the Pearson’s Chi-square test, Fisher’s exact test, or the McNemar test for paired proportions.

*VO*_2_*peak*, either expressed in mL/kg/min or as a percentage of the predicted value, was considered the primary *CPET* outcome. The remaining *CPET* parameters were considered secondary outcomes and were grouped according to their physiological domains: aerobic capacity, haemodynamic and chronotropic response, recovery kinetics, ventilatory efficiency, gas exchange, and prognostic assessment. For secondary *CPET* outcomes, *p*-values from the baseline-adjusted between-group ANCOVA analyses were adjusted for multiple comparisons using the Holm procedure within each physiological domain, controlling the family-wise error rate, and multiplicity-adjusted *p*-values were reported. In accordance with the analytical hierarchy of the study, neither absolute *VO*_2_*peak* nor predicted *VO*_2_*peak* was included in the multiplicity adjustment. Within-group pre–post comparisons were considered exploratory and their *p*-values were interpreted as nominal.

Univariate linear regression analysis was performed to evaluate the factors associated with the change in *VO*_2_*peak* from baseline to follow-up. To identify those factors independently associated with this variable, a multivariate linear regression analysis was run. To minimize data-driven model selection, a clinically prespecified model was fitted using the ENTER method, irrespective of statistical significance in univariate analyses. The model included age, sex, diabetes mellitus, ischemic etiology, heart failure duration, baseline VO_2_peak, baseline QRS duration, right heart catheterization, CRT/CRT-LBBAP, and change in left ventricular ejection fraction (ΔLVEF). Baseline pulmonary artery systolic pressure was not included in the multivariable model because of substantial missingness. Highly collinear cardiopulmonary exercise testing variables were not simultaneously entered into the model. Complete-case analysis was performed, resulting in 106 patients included in the multivariable model. Model fit was assessed using R^2^, adjusted R^2^, and the overall F-test. Multicollinearity was evaluated using tolerance and variance inflation factor (VIF), with VIF values > 5 considered indicative of potentially relevant multicollinearity. Residual assumptions were assessed using standardized residual histograms, normal probability plots, standardized residuals versus standardized predicted values, Durbin–Watson statistics, and Cook’s distance. The results are presented as unstandardized and standardized beta coefficients accompanied by their respective 95% confidence intervals.

All statistical tests were two-tailed, and a *p*-value ≤ 0.05 was defined as the threshold for statistical significance. Data processing and statistical computations were conducted using IBM SPSS Statistics, version 25.0.

## 3. Results

### 3.1. Baseline Clinical and Pharmacological Characteristics

We enrolled 111 *HFrEF* patients (median age 63 years, 69.4% male) in two cohorts: *HFimpEF* (69 patients; 62.2%) and *HFprEF* (42 patients; 37.8%). Baseline clinical and therapy measures were largely similar (Table 1). *HFimpEF* patients had a lower prevalence of diabetes (21.7% vs. 42.8%; *p* = 0.018), and less ischaemic etiology (13.0% vs. 45.2%; *p* < 0.001). *HFimpEF* patients had a shorter duration of HF (median 0 months [IQR: 0–6] vs. 11 months [IQR: 0–60]; *p* = 0.006) and a higher proportion of de novo *HF* presentation (63.8% vs. 42.8%; *p* = 0.042). Recent *HF* hospitalizations were less frequent in *HFimpEF* (≥1 admission in prior six months: 13.0% vs. 45.2%; *p* < 0.001). The median follow-up time to reassessment was 8.7 months (IQR: 7.1–12.8) for the overall cohort.

### 3.2. Ventricular Remodeling and Neurohormonal Response

Baseline measures of ventricular size, volumes, and ejection fraction were comparable between groups (Table 1). At follow-up, *HFimpEF* demonstrated greater reverse remodeling: adjusted and clinically relevant reductions in *LVEDD* (adjusted Δ −9.0 mm; 95% CI −13.8 to −4.3; *p* = 0.002; ηp^2^ = 0.248), *LVESD* (Δ −16.6 mm; 95% CI −22.7 to −9.3; *p* = 0.007; ηp^2^ = 0.518), *LVEDV* (Δ −54.1 mL; 95% CI −85.6 to −28.8; *p* < 0.001; ηp^2^ = 0.185) and *LVESV* (Δ −56.9 mL; 95% CI −80.0 to −37.1; *p* < 0.001; ηp^2^ = 0.337), with a larger adjusted increase in *LVEF* (Δ +19.9%; 95% CI 17.4–22.5; *p* < 0.001; ηp^2^ = 0.646). Other echocardiographic variables (E/e’, LA volume index, TAPSE, S’ wave) did not show significant adjusted differences (Table 2).

Also, *NT-proBNP* showed a larger adjusted decrease in *HFimpEF* (−557.6 pg/mL; 95% CI −1140 to −58.6; *p* = 0.042; ηp^2^ = 0.054), which is consistent with the structural and functional improvement.

### 3.3. Therapy Optimization

Both cohorts increased *SGLT2i* use (*HFimpEF* 53.6% to 68.1%, *p* = 0.002; *HFprEF* 40.5% to 64.3%, *p* = 0.003). Use of loop diuretics declined significantly only in *HFimpEF* (58% -> 42%, *p* = 0.019) but not in *HFprEF*. Device therapy (notably *CRT-LBBAP*) increased markedly in both groups, but was most frequent in the first one at the end of follow-up (*HFimpEF* 58.0%; *HFprEF* 35.7%; *p* = 0.023).

### 3.4. CPET Longitudinal Changes

**Figure 1:**
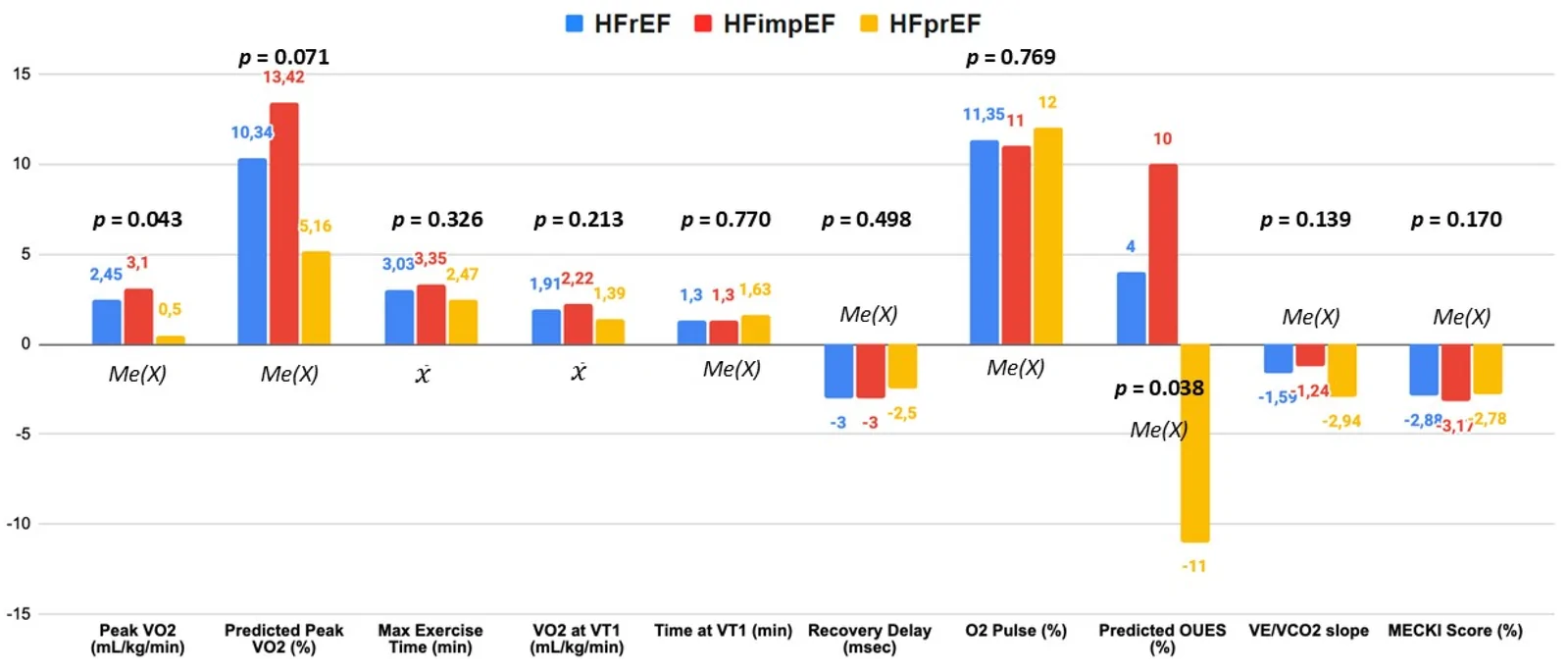
**Changes in cardiopulmonary exercise testing (CPET) parameters at follow-up according to Heart Failure phenotype**. Values represent the absolute change from baseline to the follow-up. Data are expressed as mean ($\overset{–}{x}$) or median (*MeX*) of the differences, depending on the normality of the distribution. **Abbreviations**: **VO_2_peak:** peak oxygen consumption; **Predicted PeakVO_2_%:** percentage of predicted peak oxygen consumption; **Max Exercise Time:** maximum exercise duration; **VO_2_ at VT1:** oxygen consumption at the first ventilatory threshold; **Time at VT1:** time to reach the first ventilatory threshold; **Recovery Delay:** delay in oxygen uptake recovery kinetics; **O_2_ Pulse:** oxygen pulse; **Predicted OUES:** percentage of predicted oxygen uptake efficiency slope; **VE/VCO_2_ slope:** minute ventilation/carbon dioxide production slope; **MECKI Score:** metabolic exercise cardiac kidney index.

Analysis of *CPET* parameters—assessed at baseline and following GDMT—demonstrated significant differences in functional recovery trajectories between the study cohorts Graphical Abstract, Figure 1, Table 3. The Naughton protocol was the most frequently used, being performed in 86 patients (77.5%), followed by the SOFT protocol in 11 cases (9.9%), EXTRASOFT in 9 (8.1%), and SOFT-STAND in 5 (4.5%). A total of 82 tests (73.9%) met the prespecified peak RER threshold (≥1.10) at baseline, without differences between groups (49 [71.0%] vs. 33 [78.6%], *HFimpEF* vs. *HFprEF*, *p* = 0.379). At follow-up, 83 tests (74.8%) met the prespecified peak RER, without differences between groups (49 [71.0%] vs. 34 [81.0%], improved vs. persistent reduced, *p* = 0.242).

Aerobic Capacity and Oxygen Consumption

**Figure 2:**
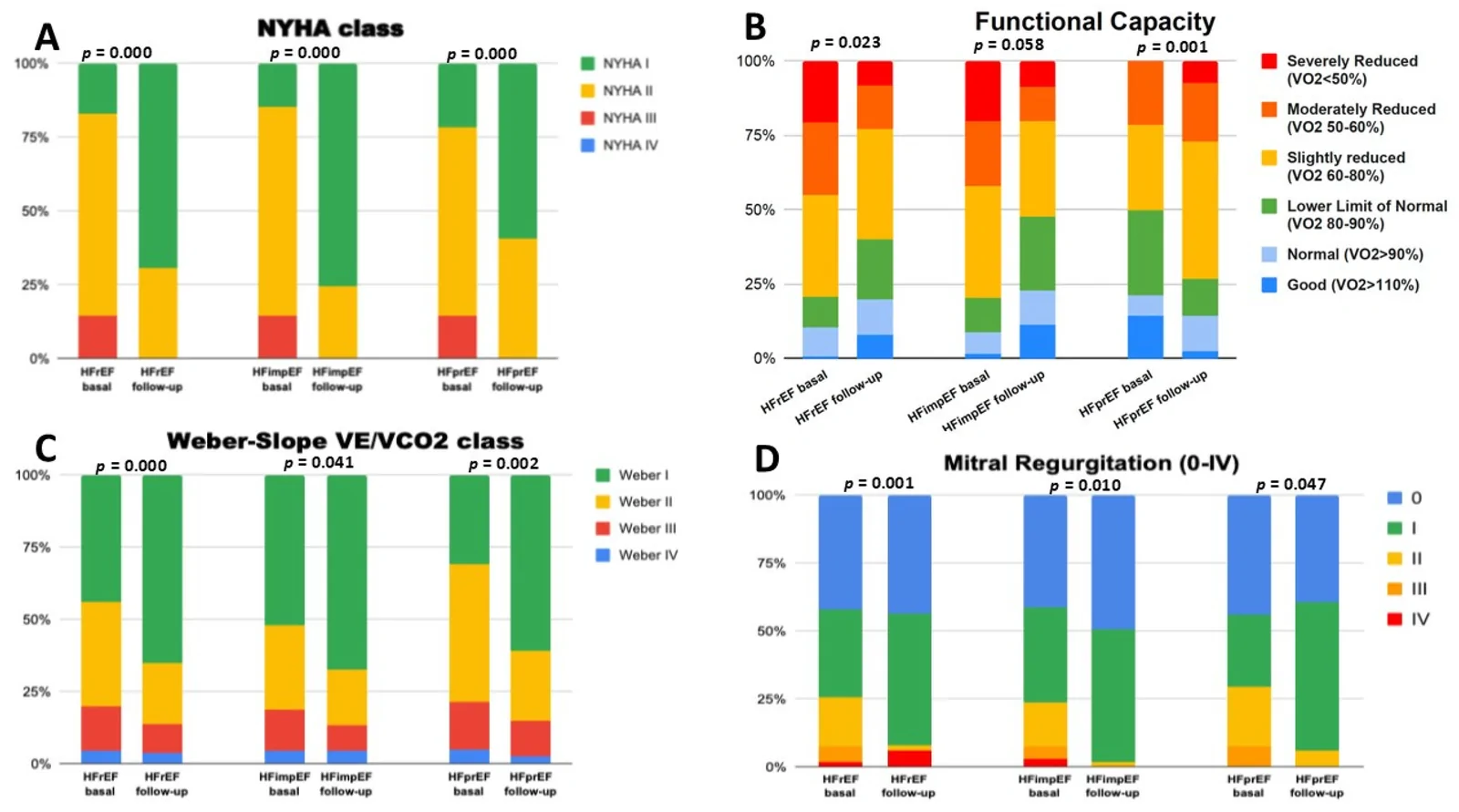
**Longitudinal Assessment of Functional and Clinical Parameters Across Heart Failure Phenotypes.** Comparative analysis of patients with *HFrEF*, *HFimpEF*, and *HFprEF* at baseline and follow-up. (**A**) Evolution of symptomatic status according to the *NYHA* Functional Class; (**B**) exercise capacity distribution based on percentage of predicted peak oxygen consumption (*VO*_2_*peak%*); (**C**) Changes in metabolic efficiency and aerobic reserve (*VE/VCO*_2_) based on the Weber Classification; (**D**) Prevalence and severity of mitral regurgitation across the study cohorts. *p*-values indicate statistical significance for intra-group functional optimization over the study period.

At baseline, both groups exhibited comparable functional impairment and were similar in terms of aerobic capacity and oxygen consumption. However, at follow-up, the *HFimpEF* group demonstrated superior improvement in *VO*_2_*peak*, from 15.5 to 19.2 mL/kg/min (*p* < 0.001, median of differences 3.1). In contrast, patients with *HFprEF* improved from 15.7 to 17.7 mL/kg/min (*p* = 0.043, median of differences 0.5). This difference between groups showed a trend by ANCOVA with bootstrapping (*p* = 0.072) and was significant by direct comparison (*p* = 0.043, Figure 1 and Table 2). This disparity became further pronounced when evaluating the percentage of predicted peak oxygen consumption (%*VO*_2_*peak*), which increased by a greater amount in *HFimpEF* (63% to 78%, *p* = 0.000, mean of differences 13.4%) than in the *HFprEF* group (60% to 68.0%, *p* = 0.167, mean of differences 5.2%), with this difference between groups being significant by ANCOVA with bootstrapping (*p* = 0.021), Table 2, and showed a trend by direct comparison (*p* = 0.071, Figure 1). In our series, moderate impairment (defined as *%VO*_2_*peak* < 60%) persisted in 20.3% of the *HFimpEF* group vs. 26.2% of *HFprEF* patients (Figure 2).

The VT1 occurred later in all phenotypes, with increased oxygen consumption. In *HFimpEF*, VO_2_-VT1 rose from 11.1 to 13.3 mL/kg/min (*p* < 0.001), while in *HFprEF* it increased from 10.9 to 12.3 mL/kg/min (*p* = 0.013). Although *HFimpEF* showed the largest absolute gains, the adjusted difference in VO_2_-VT1 between groups was 0.9 mL/kg/min (95% CI −0.2 to 1.9; *p* = 0.100; ηp^2^ = 0.023) and did not reach statistical significance after adjustment. Exercise tolerance improved overall: maximal exercise time increased by approximately 3 min across the global cohort, with *HFimpEF* reaching a mean of 13.6 min. However, the adjusted between-group difference in maximal exercise time was 1.3 min (95% CI −0.3 to 2.9; *p* = 0.104; ηp^2^ = 0.024), also not significant after adjustment.

Overall, the data show functional improvements in all groups with larger absolute gains in *HFimpEF*, but adjusted between-group comparisons did not demonstrate statistically significant differences.

Ventilatory Efficiency and Gas Exchange Kinetics

Ventilatory efficiency, a pivotal prognostic determinant in heart failure, demonstrated favorable changes across both phenotypes. The *VE/VCO*_2_ slope decreased significantly in both groups: *HFimpEF* (29.0 to 27.0; *p* < 0.0025) and *HFprEF* (33.0 to 29.6; *p* < 0.001), reflecting an optimized exertional ventilatory response and likely reduction in physiological dead space.

Regarding the *OUES*, rose significantly in *HFimpEF* (1.4 to 1.8; *p* = 0.003), but the increase in *HFprEF* (1.6 to 2.2; *p* = 0.767) was not significant. When *OUES* was normalized for age and body size (*predicted OUES%*), the trend was similar to these findings, with *HFimpEF* nearing normal values (97% ± 29%; *p* = 0.052), Figure 1. However, adjusted ANCOVA comparisons revealed no significant between-group differences at follow-up for either *VE/VCO*_2_ slope (*p* = 0.755) or predicted OUES% (*p* = 0.077), Table 3.

Hemodynamic Parameters, Cardiac Reserve and Recovery Kinetics

The oxygen pulse -a surrogate measure of stroke volume during exertion- showed substantial improvement in both cohorts, with a more pronounced absolute gain in *HFimpEF* patients (from 10.1 to 12.3 mL/beat, +2.08; *p* < 0.001) than in *HFprEF* (+1.5 mL/beat; *p* = 0.001). Notably, when expressed as a percentage of predicted value, oxygen pulse reached near-normal levels in the *HFimpEF* group (75% to 89%; *p* = 0.008), whereas the increase in *HFprEF* did not achieve statistical significance. Although between-group ANCOVA comparisons at follow-up showed no significant differences for oxygen pulse (*p* = 0.239), post-exercise recovery dynamics revealed a distinct physiological advantage for *HFimpEF*. Specifically, *Recovery Delay* showed a nominal between-group difference favoring *HFimpEF* (adjusted difference: −9.0 ms; 95% CI: −18.0 to −0.6; nominal *p* = 0.047). However, after adjustment for multiple comparisons within the recovery kinetics domain using the Holm procedure, this association was no longer statistically significant (Holm-adjusted *p* = 0.141). Accordingly, *Recovery Delay* should be considered an exploratory finding rather than a confirmatory between-group difference. This comprehensive functional optimization was accompanied by a marked improvement in clinical prognosis, as assessed by the *MECKI* score [6], which decreased significantly in both *HFimpEF* [3.9% to 0.6%; *p* < 0.001] and *HFprEF* [5.9% to 3.3%; *p* < 0.001], without significant differences between groups.

### 3.5. Variables Associated with VO_2_peak Response at Follow-Up

In the univariate analysis, the following were identified as associated with *VO*_2_*peak* improvement at follow-up: diabetes mellitus, baseline PASP, baseline *VO*_2_*peak*, baseline *%predictedVO*_2_*peak*, VO_2_-VT1, %predicted VO_2_-VT1, exercise duration, baseline O_2_ pulse, baseline %predicted O_2_ pulse, and baseline QRS duration. In the prespecified multivariable linear regression analysis, 106 patients with complete data were included. The overall model was statistically significant (F(10,95) = 4.919, *p* < 0.001), explaining 34.1% of the variability in the change in VO_2_peak (R^2^ = 0.341; adjusted R^2^ = 0.272). Older age (B = −0.118 mL/kg/min per year; 95% CI, −0.207 to −0.029; standardized β = −0.261; 95% CI, −0.458 to −0.064; *p* = 0.010), diabetes mellitus (B = −2.250 mL/kg/min; 95% CI, −4.190 to −0.310; standardized β = −0.210; 95% CI, −0.392 to −0.029; *p* = 0.023), and higher baseline VO_2_peak (B = −0.580 mL/kg/min per mL/kg/min; 95% CI, −0.774 to −0.387; standardized β = −0.569; 95% CI, −0.759 to −0.379; *p* < 0.001) were independently associated with a lower magnitude of VO_2_peak improvement. No statistically significant independent associations were observed for sex, ischemic etiology, heart failure duration, baseline QRS duration, right heart catheterization, CRT-LBBAP, or ΔLVEF. In particular, greater ΔLVEF was not independently associated with ΔVO_2_peak (B = 0.019; 95% CI, −0.058 to 0.096; standardized β = 0.052; *p* = 0.617). No VIF exceeded 5, although relatively higher values were observed for baseline QRS duration (VIF = 4.021) and CRT/CRT-LBBAP (VIF = 4.848). Residual diagnostics did not identify major departures from the regression assumptions. Standardized residuals ranged from −2.017 to 3.324, and the maximum Cook’s distance was 0.177, suggesting no highly influential individual observation, Table 4.

## 4. Discussion

The *IMPRO_2_VEMENT*-*HF* study demonstrates that comprehensive management of *HFrEF* yields substantial and multifaceted gains in functional capacity and cardiopulmonary efficiency across the entire patient spectrum; our findings reveal a partial decoupling between echocardiographic improvement and exertional adaptations: while both *HFimpEF* and *HFprEF* phenotypes exhibited significant intra-group improvements in aerobic performance, ventilatory efficiency, and prognostic risk scores, between-group adjusted ANCOVA comparisons revealed that the magnitude of overall functional trajectory was largely comparable between phenotypes. Rather than displaying a complete divergence, these findings indicate that baseline *HFrEF* patients derive meaningful exertional benefits from contemporary guideline-directed therapies regardless of the extent of *LVEF* recovery, though specific metrics -such as normalized peak oxygen consumption (%predicted VO2peak) highlight a subtle, distinct advantage in those achieving reverse left ventricular remodeling.

Improvement in exercise capacity and Oxygen Consumption. Predictors of *VO*_2_*peak* Improvement.

The primary finding of our study lies in the gain in *VO*_2_*peak* observed within the *HFimpEF* cohort. Although a global improvement in aerobic capacity was noted across the entire study population, the *HFimpEF* subgroup achieved a median increase of 3.0 mL/kg/min, which substantially exceeded the 0.5 mL/kg/min median gain reported in the *HFprEF* group. These findings align with prior large-scale registry data, notably the studies by Agostoni et al. [12], based on the MECKI score database, and Magrí et al. [15]. Agostoni et al. documented significantly higher absolute *VO*_2_*peak* values in *HFimpEF* compared to persistent *HFprEF* (15.5 vs. 14.0 mL/kg/min; or 62.2% vs. 52.6% of predicted), while Magrí et al. reported a similar superiority in recovered mid-range *EF* patients (16.1 vs. 14.4 mL/kg/min; or 63% vs. 53% of predicted). The consistency between both studies and its relationship with our data is notable. Both place peak oxygen consumption at around 16.5–16.7 mL/kg/min for the recovered group. These studies validate that the recovery of *LVEF* typically leads to a gain of approximately 2.5 to 3 mL/kg/min in aerobic capacity compared to patients who do not improve, even though the increase in our series was somewhat smaller (+1.7 mL/kg/min). However, a crucial methodological distinction must be emphasized when contextualizing these comparisons. Both Agostoni et al. and Magrí et al. evaluated cross-sectional baseline differences between *EF* phenotypes without performing an adjusted ANCOVA analysis to compare the longitudinal magnitude of change between groups over time (Table S1). When we applied and adjusted ANCOVA model to account for baseline differences, the net between-group difference in absolute *VO*_2_*peak* gain did not reach statistical significance, whereas the percentage of predicted *VO*_2_*peak* did demonstrate a significant adjusted superior recovery in *HFimpEF* (adjusted difference: 8.5%; 95% CI: 1.2 to 16.1; *p* = 0.021). This suggests that while absolute aerobic gains reflect a broad therapeutic response across *HFrEF* phenotypes, normalized functional capacity (%predicted *VO*_2_*peak*) represents a more sensitive marker of the distinct metabolic advantage associated with *LVEF* recovery.

Time to VT1 represents the functional reserve for daily activities, such as walking, climbing a flight of stairs, or dressing, which occur at intensities near the first ventilatory threshold. In heart failure, not only is the “pump” function impaired, but skeletal muscles also exhibit lower mitochondrial density and reduced blood flow, causing the patient to reach the anaerobic threshold (VT1) much sooner than normal. Conversely, one might hypothesize that patients who improve their ejection fraction would delay the time to the first threshold. Following our comprehensive therapeutic intervention, we observed a significant delay in the time to *VT1* across the entire study population. Notably, this improvement was virtually identical between the *HFimpEF* and *HFprEF* cohorts (ANCOVA *p* = 0.975). Rather than being solely dependent on *LVEF* recovery, this parallel gain indicates that peripheral muscular adaptations and the optimization of submaximal aerobic metabolism can be successfully achieved regardless of the extent of central pump restoration.

Furthermore, the enhancement in exercise tolerance -manifested by an increase in peak exercise duration (+3.4 min in *HFimpEF* vs. +2.5 min in *HFprEF*) -underscores the clinical impact of a comprehensive therapeutic approach. This multifaceted intervention, integrating *GDMT*, *CRT-LBBAP*, and structured *CRP*, directly contributes to superior improvements in patient quality of life.

A key finding of our study is the identification of specific clinical characteristics associated with the magnitude of functional recovery. To the best of our knowledge, there is limited evidence in the current literature regarding the determinants of longitudinal improvement in *VO*_2_*peak* in this specific patient population. While many studies have focused on the prognostic value of absolute *VO*_2_*peak* values [5,7,15,16,17], our data provide a novel perspective by analyzing the clinical factors associated with the magnitude of improvement in aerobic capacity over time.

In the prespecified multivariable analysis, older age, diabetes mellitus, and higher baseline VO_2_peak were independently associated with a lower magnitude of functional improvement. The inverse association with baseline VO_2_peak suggests a possible “ceiling effect”, whereby patients starting with a higher aerobic capacity have less margin for substantial improvement than those with greater baseline functional impairment. This interpretation is consistent with previous observations showing that patients with lower baseline exercise capacity may experience larger gains following optimization of heart failure treatment and cardiac resynchronization [18,19,20]. Diabetes mellitus was also independently associated with smaller improvements in VO_2_peak, possibly reflecting the combined influence of metabolic abnormalities, microvascular dysfunction, skeletal muscle impairment, and reduced physiological adaptability to exercise.

Baseline QRS duration showed a positive association with VO_2_peak improvement in the univariate analysis; however, this association was not maintained after adjustment for the prespecified clinical and therapeutic covariates. Therefore, our findings do not support baseline QRS duration as an independent determinant of functional improvement in this cohort. This result should be interpreted in the context of the complex relationship between electrical conduction delay, ventricular dyssynchrony, and response to cardiac resynchronization. Although prolonged QRS duration is an established marker used in the selection of patients for resynchronization therapy and has been associated with response to CRT-related outcomes, QRS duration alone does not directly quantify mechanical dyssynchrony and may not consistently predict changes in exercise capacity. Accordingly, the unadjusted association observed in our cohort may reflect, at least in part, differences in the underlying substrate and in the likelihood of receiving resynchronization therapy rather than an independent effect of QRS duration itself.

Analysis of ventilatory efficiency and hemodynamic response to exercise

Beyond aerobic capacity, ventilatory efficiency -quantified by the *VE/VO*_2_ *slope*- serves as a critical prognostic indicator in heart failure [14]. In the current study, the *HFimpEF* group demonstrated a statistically significant reduction in this parameter, decreasing from a baseline of 29 to 27. Clinically, a follow-up value of 27 reclassifies these patients within a low-risk stratum (Weber Class A), outperforming the efficiency levels reported by Agostoni et al. [12], who documented a mean slope of 30.6 in the *MECKI* score database. Regarding *OUES*, at baseline, it was depressed across all groups, reflecting a systemic impairment in the efficiency of oxygen extraction and utilization. Our findings regarding the significant increase in *OUES* within the *HFimpEF* cohort align with the large-scale observations from the *MECKI* score data base reported by Agostoni et al. and Magrì et al. [12,15]. These authors emphasized that physiological recovery in *HF* patients is multifaceted, involving not just *LVEF* improvement but also a significant shift in ventilatory efficiency and oxygen uptake kinetics. Specifically, the improvement of *OUES* in our *HFimpEF* group reinforces the concept that recovered patients achieve a more efficient metabolic response to exercise, which is traditionally associated with a better long-term prognosis in the literature. However, a crucial statistical distinction arises when comparing our longitudinal methodology with previous registry data. While both Agostoni et al. and Magrí et al. established significant baseline differences between *EF* phenotypes using cross-sectional univariate comparisons (e.g., Mann–Whitney U or *t*-tests), neither study performed an adjusted ANCOVA analysis to account for baseline imbalances when evaluating functional changes over time. In our series, when evaluating these ventilatory efficiency parameters in adjusted between-group ANCOVA models, the net differences at follow-up between both groups did not reach statistical significance (Table S1). This lack of statistical divergence after ANCOVA adjustment indicates that exertional ventilatory optimization is a shared, global benefit of modern, comprehensive heart failure therapy across *HFrEF* phenotypes, rather than an exclusive physiological feature driven solely by *LVEF* recovery.

Concurrently, a notable normalization of the oxygen pulse was observed, with a higher proportion seen in the *HFimpEF* cohort, which reflects a more efficient stroke volume during exercise; given that oxygen pulse is a surrogate for stroke volume and cardiac output during exertion, these data suggest that *LVEF* recovery is coupled with a restoration of hemodynamic reserved, facilitating more efficient cardiac performance during physical stress. In our series, the proportion of patients who achieved a normalization of the %predicted oxygen pulse was higher than that reported by Agostoni et al. [12], which was calculated at 62.2% and 52.6% for *HFimpEF* and *HFprEF*, respectively.

Reverse remodeling and metabolic efficiency

The debate regarding whether a heart with recovered *LVEF* represents a truly “cured” state remains complex. Our findings demonstrate a significant reduction in ventricular volumes and NT-proBNP levels within the *HFimpEF* cohort (from 1202 to 372 pg/mL). However, existing literature warns against the “echocardiographic recovery false positive”: if *LVEF* improvement is not accompanied by objective functional gains (e.g., *VO*_2_*peak* > 55% of predicted), a substantial residual risk for cardiovascular events persists. In fact, although our study did not include an analysis of *CPET* predictors for cardiovascular events, it is worth noting that up to one-fifth of patients who show improvement in their *LVEF* still exhibit moderately impaired functional capacity (*predicted %VO*_2_ < 60%).

In the *IMPRO*_2_*VEMENT-HF* study, functional optimization directly translated into an improved clinical prognosis as projected by the *MECKI* score, with a more pronounced risk reduction in the *HFimpEF* group. Consequently, *HF* risk is not static; therefore, *HFrEF* patients should have follow-up *CPETs* to restratify their risk, allowing for early detection of relapses and optimized exercise capacity through tailored pharmacological therapy and exercise [21].

Several limitations must be acknowledged. First, the retrospective nature of the study may introduce inherent selection biases. Second, the sample size is relatively modest when compared to large-scale registries, such as the *MECKI* score database, and may have limited the precision and stability of the multivariable regression estimates. Nevertheless, to minimize the risk of data-driven model selection, the multivariable analysis was based on a clinically prespecified set of variables selected according to clinical relevance and previous evidence rather than univariate statistical significance. Finally, as a single-center study, the generalizability of our findings to other clinical settings may be limited. Nonetheless, the meticulous depth of the *CPET* data provides robust internal validity, lending significant weight to our conclusions.

## 5. Conclusions

Comprehensive *HFrEF* management yields substantial functional gains across the entire patient spectrum. Although *HFimpEF* patients achieve superior gains in normalized aerobic capacity (%predicted *VO*_2_*peak*), adjusted comparisons reveal no significant between-group differences in absolute peak *VO*_2_, submaximal exercise thresholds, ventilatory efficiency or recovery kinetics. This partial decoupling between central structural remodeling and functional recovery underscores that residual exertional limitations persist despite *LVEF* normalization. *CPET* may be a useful tool for longitudinally assessing functional recovery and risk stratification in these patients.

## Figures and Tables

**Table 1:** **Baseline clinical characteristics**.

	HFrEF (N = 111)	HFimpEF (N = 69)	HFprEF (N = 42)	*p*
Male sex (%)	77/111 (69.4%)	47/69 (68.1%)	30/42 (71.4%)	0.713 (†)
Age (years)	63 (56–69)	63 (57–69.5)	63 (55–67)	0.388
CV risk factor				
DM	33/111 (29.7%)	15/69 (21.7%)	18/42 (42.8%)	0.018 (†)
HTN	57/111 (51.4%)	32/69 (46.4%)	25/42 (59.5%)	0.179 (†)
DLP	34/111 (30.6%)	19/69 (27.5%)	15/42 (35.7%)	0.365 (†)
Smoking	19/110 (17.3%)	12/69 (17.4%)	7/41 (17.1%)	0.939 (†)
≥1 HA for HF in the last 6 months	28/111 (25.2%)	9/69 (13.0%)	19/42 (45.2%)	<0.001 (†)
New-onset heart failure	62/110 (56.4%)	44/69 (63.8%)	18/41 (43.9%)	0.042 (†)
HF evolution time (months)	0 (0–14)	0 (0–6)	11 (0–60)	0.006
NYHA				
I–II	95/111 (85.5%)	59/69 (85.5%)	36/42 (85.7%)	0.976 (†)
III–IV	16/111 (14.4%)	10/69 (14.5%)	6/42 (14.3%)	
Ischaemic Etiology	28/111 (25.2%)	9/69 (13.0%)	19/42 (45.2%)	<0.001 (†)
Atrial Fibrillation	25/110 (22.7%)	18/68 (26.5%)	7/42 (16.7%)	0.469 (†)
QRS (ms)	134 (110–175)	146 (100–176)	112 (110–169)	0.106
QRS > 120 ms				
LBBB	60/111 (54.1%)	41/69 (59.4%)	19/42 (45.2%)	0.052 (†)
RBBB	5/111 (4.5%)	3/69 (4.3%)	2/42 (4.8%)	
LVEF (%)	30 (24–35)	31 (24–35)	29.5 (22–35)	0.482
LVEDD (mm)	60.9 ± 9.4	60.4 ± 8.4	61.3 ± 10.1	0.660
LVESD (mm)	53.5 ± 10.4	53.7 ± 11.4	53.2 ± 8.7	0.346
LVEDV (mL)	165 (129–214)	168 (124–208)	159 (133–220)	0.971
LVESV (mL)	116 (85–153)	121 (84–153)	112 (92–163)	0.990
TAPSE (mm)	20 (18–24)	21.5 (18–24)	20 (17–23.5)	0.263
S’ (cm/s)	10.5 ± 2.6	10.7 ± 2.6	10.1 ± 2.7	0.302
LAVI (ml/m^2^)	32 (25–45)	29 (23–32)	32 (27–47)	0.099
PASP (mmHg)	24 (20–32)	22 (19–30)	28 (22–34)	0.102
Cr (mg/dL)	1.0 ± 0.3	1.0 ± 0.3	1.1 ± 0.3	0.918
GFR (mL/min)	72 (60–89)	72 (59–87)	74 (60–89)	0.867
NT-proBNP (pg/mL)	1217 (527–2672)	1202 (506–2659)	1295 (529–2711)	0.375
Hb (g/dL)	14.2 ± 1.4	14.3 ± 1.4	14.2 ± 1.4	0.794
Ferritin (ug/L)	104 (48–249)	124 (56–249)	99.5 (37–228)	0.485
TSI (%)	25 (17–33)	25 (18–32)	23 (14–35)	0.346
Treatment:				
ACO	28/110 (25.5%)	18/68 (26.5%)	10/42 (23.8%)	0.756 (†)
ACEI/ARB/ARNI	110/111 (99.1%)	68/69 (98.5%)	42/42 (100%)	1.000 (‡)
βB	108/111 (97.3%)	66/69 (95.7%)	42/42 (100%)	0.288 (‡)
Loop diuretics	66/111 (59.4%)	40/69 (58.0%)	26/42 (61.9%)	0.682 (†)
MRA	93/111 (83.8%)	59/69 (85.5%)	34/42 (81.0%)	0.528 (†)
SGLT2i	54/111 (48.6%)	37/69 (53.6%)	17/42 (40.5%)	0.179 (†)
ICD	5/111 (4.5%)	0	5 (11.9%)	0.007 (‡)
CRT-LBBAP	2/111 (1.8%)	1/69 (1.4%)	1/42 (2.4%)	1.000 (‡)

**Abbreviations: ACEI,** Angiotensin-Converting Enzyme Inhibitor; **ACO,** Oral Anticoagulation; **ARB,** Angiotensin II Receptor Blocker; **ARNI,** Angiotensin Receptor-Neprilysin Inhibitor; **βB,** Beta-blocker; **Cr,** Creatinine; **CRT-LBBAP,** Cardiac Resynchronization Therapy via Left Bundle Branch Area Pacing; **CV,** Cardiovascular; **DLP,** Dyslipidemia; **DM,** Diabetes Mellitus; **GFR,** Glomerular Filtration Rate; **HA for HF,** Hospital Admission for Heart Failure; **Hb,** Hemoglobin; **HFimpEF,** Heart Failure with improved Ejection Fraction; **HFrEF,** Heart Failure with reduced Ejection Fraction; **HFprEF,** Heart Failure with persistent reduced Ejection Fraction; **HTN,** Hypertension; **ICD,** Implantable Cardioverter Defibrillator; **LAVI,** Left Atrial Volume Index; **LBBB,** Left Bundle Branch Block; **LVEDD,** Left Ventricular End-Diastolic Diameter; **LVEDV,** Left Ventricular End-Diastolic Volume; **LVEF,** Left Ventricular Ejection Fraction; **LVESD,** Left Ventricular End-Systolic Diameter; **LVESV,** Left Ventricular End-Systolic Volume; **MRA,** Mineralocorticoid Receptor Antagonist; **NT-proBNP,** N-terminal pro-B-type Natriuretic Peptide; **NYHA,** New York Heart Association; **PASP,** Pulmonary Artery Systolic Pressure; **RBBB,** Right Bundle Branch Block; **S’ wave,** Systolic Mitral Annular Velocity; **SGLT2i,** Sodium-Glucose Cotransporter-2 Inhibitor; **TAPSE,** Tricuspid Annular Plane Systolic Excursion; **TSI,** Transferrin Saturation Index; **Vol AI,** Left Atrial Volume. (**†**) *p*-value obtained using Pearson`s Chi-Square test; (**‡**) *p*-value obtained using Fisher’s exact test.

**Table 2:** Baseline clinical, biochemical, echocardiographic and therapeutic characteristics of the study population. Evolution of clinical, laboratory, echocardiographic and therapeutic parameters from baseline to follow-up across heart failure phenotypes. Continuous variables are expressed as mean ± standard deviation or median [interquartile range], and categorical variables as absolute numbers and percentages.

	HFrEF (N = 111)			HFimpEF (N = 69)			HFprEF (N = 42)			ANCOVA (Bootstrap) (†)			
	Baseline	Follow-Up	*p*-Value	Baseline	Follow-Up	*p*-Value	Baseline	Follow-Up	*p*-Value	Adjusted Difference at Follow-Up	95% CI	*p*-Value	Partial Square Eta
Laboratory parameters													
*NTproBNP* (pg/mL)	1217 (527–2672)	486 (173–1136)	0.000	1202 (506–2659)	372 (142–835)	0.000	1295 (529–2711)	787 (375–1955)	0.004	−557.6	−1140–(−58.6)	0.042	0.054
*Hb* (g/dL)	14.2 ± 1.4	14.7 ± 1.6	0.001	14.3 ± 1.4	14.6 ± 1.4	0.080	14.2 ± 1.4	14.9 ± 1.8	0.004	−0.4	−0.8–0.1	0.142	0.020
*Ferritin* (microg/L)	104 (48–249)	79 (37–165)	0.000	124 (56–249)	94 (36.7–171)	0.000	99.5 (37–228)	65 (33–154)	0.003	−9.7	−48.2–34.2	0.600	0.004
*TSI* (%)	25 (17–33)	25 (18–32)	0.692	25 (18–32)	24 (18–32)	0.906	23 (14–35)	26 (19–33)	0.489	−2.1	−7.3–3.4	0.411	0.009
*Creatinine* (mg/dL)	1.0 ± 0.3	1.0 ± 0.3	0.394	1.0 ± 0.3	1.0 ± 0.4	0.669	1.1 ± 0.3	1.0 ± 0.3	0.434	−0.01	−0.10–0.08	0.752	0.001
*GFR* (mL/min)	72 (60–89)	77 (63.5–88)	0.193	72 (59–87)	73 (63–88)	0.636	74 (60–89)	79 (63–89.5)	0.152	−2.5	−8.7–3.2	0.443	0.007
Echocardiography													
*LVEF* (%)	30 (24–35)	47 (38–58)	0.000	31 (24–35)	56 (47.5–60.5)	0.000	29.5 (22–35)	35 (31–40)	0.000	+19.9	17.4–22.5	0.000	0.646
*LVEDD* (mm)	60.9 ± 9.4	55.7 ± 8.4	0.000	60.4 ± 8.4	53.3 ± 6.4	0.000	61.3 ± 10.1	61.9 ± 9.5	0.964	−9.0	−13.8–(−4.3)	0.002	0.248
*LVESD* (mm)	53.5 ± 10.4	40.2 ± 8.5	0.000	53.7 ± 11.4	37.0 ± 6.9	0.001	53.2 ± 8.7	49.4 ± 5.3	0.007	−16.6	−22.7–(−9.3)	0.007	0.518
*LVEDV* (mL)	165 (129–214)	116 (89–150)	0.000	168 (124–208)	103 (86–133)	0.000	159 (133–220)	145.5 (112.5–198)	0.028	−54.1	−85.6–(−28.8)	0.000	0.185
*LVESV* (mL)	116 (85–153)	57 (41–83)	0.000	121 (84–153)	46 (34–59)	0.000	112 (92–163)	87.5 (72–132.5)	0.008	−56.9	−80.0–(−37.1)	0.000	0.337
*E/e’*	11.5 ± 4.7	9.4 ± 3.8	0.002	11.5 ± 4.9	8.6 ± 3.0	0.031	11.5 ± 4.5	11.2 ± 4.5	0.807	−2.3	−4.6–(−0.03)	0.060	0.078
*E/A*	0.8 (0.6–1.9)	0.9 (0.7–1.4)	0.846	0.8 (0.6–1.2)	1.0 (0.8–1.5)	0.056	0.9 (0.6–2.2)	0.8 (0.6–1.5)	0.134	+0.19	−1.1–1.2	0.748	0.003
*LAVI* (mL/m^2^)	32 (25–45)	33 (23–41)	0.132	29 (23–32)	30 (21–38)	0.025	32 (27–47)	36 (32–47)	0.657	−3.8	−10.5–3.4	0.400	0.005
*TAPSE* (mm)	20 (18–24)	21 (19–25)	0.229	21.5 (18–24)	22 (19–24)	0.675	20 (17–23.5)	21 (18–25)	0.109	−0.5	−2.4–1.5	0.623	0.003
*PASP* (mmHg)	24 (20–32)	22 (19–27)	0.121	22 (19–30)	21 (18.5–24)	0.054	28 (22–34)	26 (19.5–30.5)	0.917	−5.8	−9.7–(−1.8)	0.008	0.204
*S*’ *wave* (cm/s)	10.5 ± 2.6	11.7 ± 3.6	0.023	10.7 ± 2.6	11.9 ± 4.2	0.059	10.1 ± 2.7	11.4 ± 1.9	0.279	+0.3	−2.8–2.8	0.814	0.001
Treatment												*p*-value (*)	
*ACEI/ARB/ARNI*	110 (99.1%)	107 (96.4%)	1.00	68 (98.5%)	67 (97.1%)	1.000	42 (100%)	40 (95.2%)	1.000			0.715	
*Betablockers*	108 (97.3%)	104 (93.7%)	0.687	66 (95.7%)	64 (92.8%)	1.000	42 (100%)	40 (95.2%)	1.000			0.405	
*Loop diuretics*	66 (59.4%)	49 (44.1%)	0.008	40 (58.0%)	29 (42.0%)	0.019	26 (61.9%)	20 (47.6%)	0.267			0.459	
*MRA*	93 (83.8%)	95 (85.6%)	0.549	59 (85.5%)	61 (88.4%)	0.625	34 (81.0%)	34 (81.0%)	1.000			0.305	
*SGLT2i*	54 (48.6%)	74 (66.7%)	0.000	37 (53.6%)	47 (68.1%)	0.002	17 (40.5%)	27 (64.3%)	0.003			0.861	
*ICD*	5 (4.5%)	20 (18.0%)	0.000	0	6 (8.7%)	0.001	5 (11.9%)	14 (33.3%)	0.004			0.001	
*CRT-LBBAP*	2 (1.8%)	55 (49.5%)	0.000	1 (1.4%)	40 (58.0%)	0.000	1 (2.4%)	15 (35.7%)	0.000			0.023	
*CRP*	0	41 (36.9%)	--	0	24 (34.8%)	--	0	17 (40.5%)	--			0.483	

**Abbreviations: ACEI**, Angiotensin-Converting Enzyme Inhibitor; **ARB**, Angiotensin II Receptor Blocker; **ARNI**, Angiotensin Receptor-Neprilysin Inhibitor; **CRP,** Cardiac Rehabilitation Program; **CRT-LBBAP**, Cardiac Resynchronization Therapy via Left Bundle Branch Area Pacing; **E/A**, Ratio of transmitral early to late left ventricular filling velocity; **E/e’**, Ratio of early transmitral flow velocity to early diastolic annular velocity; **GFR**, Glomerular Filtration Rate; **Hb**, Hemoglobin; **ICD**, Implantable Cardioverter Defibrillator; **LAVI**, Left Atrial Volume Index; **LVEDD**, Left Ventricular End-Diastolic Diameter; **LVEDV**, Left Ventricular End-Diastolic Volume; **LVEF**, Left Ventricular Ejection Fraction; **LVESD**, Left Ventricular End-Systolic Diameter; **LVESV**, Left Ventricular End-Systolic Volume; **MRA**, Mineralocorticoid Receptor Antagonist; **NTproBNP**, N-terminal pro-B-type Natriuretic Peptide; **PASP**, Pulmonary Artery Systolic Pressure; **S’ wave**, Peak Systolic Mitral Annular Velocity; **SGLT2i**, Sodium-Glucose Cotransporter-2 Inhibitor; **TAPSE**, Tricuspid Annular Plane Systolic Excursion; **TSI**, Transferrin Saturation Index; **(†) ANCOVA (Bootstrap) model** compares the follow-up values between *HFimpEF* and *HFprEF* groups, using the baseline value of each variable as a covariate. Adjusted difference at follow-up, 95% Confidence Interval (95% CI), *p*-value, and Partial eta squared are based on 2000 bootstrap samples (BCa method). (*) Treatments are categorical variables and were excluded from the ANCOVA model. The *p*-value in these variables was obtained by the *Chi-square test* and compares the proportion of treatment prescriptions at last follow-up.

**Table 3:** **Cardiopulmonary exercise testing parameters across heart failure phenotypes**. Comparative analysis of gas exchange, ventilatory efficiency, and hemodynamic response during maximal exercise. Data are presented as mean ± standard deviation or median [interquartile range], as appropriate.

	HFrEF (N = 111)			HFimpEF (N = 69)			HFprEF (N = 42)				ANCOVA (Bootstrap) (†)			
	Baseline	Follow-Up	*p*-Value	Baseline	Follow-Up	*p*-Value	Baseline	Follow-Up	*p*-Value	Adjusted Difference at Follow-Up	95% CI	*p*-Value	Multiplicity-Adjusted *p*-Value	Partial Square Eta
CPET														
Time interval between baseline and follow-up CPET (months)	10.3 ± 4.5			10.1 ± 4.4			10.7 ± 4.7							
Main Outcome														
*VO*_2_*peak* (mL/kg/min)	15.5 ± 4.7	18.6 ± 5.1	0.000	15.5 ± 5.2	19.2 ± 5.5	0.000	15.7 ± 3.9	17.7 ± 4.5	0.043	+1.6	−0.1–3.2	0.072		0.030
*Predicted VO*_2_*peak* (%)	63 (IQR 54–78)	74.5 (IQR 60–87)	0.000	63 (IQR 54–78.5)	78 (IQR 66–88)	0.000	60 (IQR 52.5–77.3)	68 (IQR 58–82)	0.167	+8.5	1.2–16.1	0.021		0.041
**Aerobic capacity**														
*VO*_2_*at VT1* (mL/kg/min)	11.0 ± 3.4	12.9 ± 3.2	0.000	11.1 ± 3.3	13.3 ± 3.5	0.000	10.9 ± 2.7	12.3 ± 2.5	0.013	+0.9	−0.2–1.9	0.100	0.400	0.023
*% Predicted VO*_2_*at VT1* (%)	46 ± 15	53 ± 17	0.000	45 ± 15	56 ± 18	0.000	48 ± 14	50 ± 15	0.476	−11.4	−59.3–34.6	0.642	1.000	0.002
*Max Exercise Time* (min)	10.6 ± 5.2	13.7 ± 5.4	0.000	10.2 ± 5.0	13.6 ± 5.7	0.000	11.3 ± 5.5	13.8 ± 5.1	0.002	+1.3	−0.3–2.9	0.104	0.400	0.024
*Time at VT1* (min)	4.9 ± 2.7	6.3 ± 2.7	0.000	4.8 ± 2.5	6.3 ± 2.6	0.002	4.9 ± 3.1	6.4 ± 2.8	0.001	0.0	−1.1–1.1	0.975	1.000	0.000
Haemodynamic and chronotropic response														
*Peak HR* (bpm)	126 ± 22	126 ± 20	0.410	129 ± 23	128 ± 20.0	0.552	123 ± 20	123 ± 19	0.581	+3.0	−4.0–9.5	0.400	1.000	0.006
*HR at VT1* (bpm)	102 ± 19	98 ± 17	0.087	105 ± 20	100 ± 17	0.229	98 ± 16	95 ± 15	0.195	+4.7	−0.9–10.4	0.124	0.620	0.022
*Resting HR* (bpm)	75 (IQR 68–82)	71 (IQR 64–81)	0.052	76 (IQR 69–88)	70 (IQR 61–81)	0.076	73 (IQR 65–80)	73 (IQR 66–81)	0.397	+0.4	−3.9–4.8	0.870	1.000	0.000
*O*_2_*Pulse* (mL/beat)	10.4 ± 3.6	12.3 ± 4.1	0.000	10.1 ± 3.9	12.3 ± 4.3	0.000	10.8 ± 3.2	12.3 ± 3.8	0.001	+0.3	−0.9–1.5	0.582	1.000	0.003
*O*_2_*Pulse* (%)	74 ± 19	87.2 ± 29.1	0.001	75 ± 21	89 ± 32	0.008	73 ± 18	84 ± 24	0.068	+6.9	−3.9–17.2	0.239	0.956	0.014
Recovery kinetics														
*Peak RER* (ratio)	1.1 ± 0.2	1.1 ± 0.2	0.065	1.1 ± 0.1	1.1 ± 0.1	0.135	1.1 ± 0.1	1.1 ± 0.1	0.273	0.0	−0.1–0.1	0.955	0.955	0.000
*RER at 2 min recovery* (ratio)	1.2 ± 0.1	1.2 ± 0.1	0.704	1.2 ± 0.2	1.2 ± 0.2	0.95	1.2 ± 0.1	1.2 ± 0.1	0.571	0.0	−0.1–0.0	0.469	0.938	0.005
*Recovery Delay* (ms)	31 (IQR 14–50)	30 (IQR 10–40)	0.071	30 (IQR 10–50)	29 (IQR 10–40)	0.057	35 (IQR 22–50)	30 (IQR 10–40)	0.601	−9.0	−18.0–(−0.6)	0.047	0.141	0.072
Ventilatory efficiency														
*OUES*	1.5 (IQR 0.9–2.3)	1.9 (IQR 1.4–2.5)	0.006	1.4 (IQR 0.9–1.7)	1.8 (IQR 1.5–2.4)	0.003	1.6 (IQR 1.1–3.0)	2.2 (IQR 1.4–2.8)	0.767	−0.8	−2.9–0.7	0.491	1.000	0.028
*Predicted OUES* (%)	89 ± 32.6	96 ± 29	0.240	87 ± 33	97 ± 29	0.052	97 ± 32	92 ± 28	0.107	+19.6	−3.2–41.7	0.077	0.385	0.073
*VE/VCO*_2_ *slope*	31 (IQR 27–35)	27.9 (IQR 25.3–32.0)	0.000	29.0 (IQR 26–34)	27.0 (IQR 24.9–31.4)	0.025	33 (IQR 29–36)	29.6 (IQR 26.3–33.0)	0.000	−0.4	−2.6–2.2	0.755	1.000	0.001
*VE/VCO*_2_*at VT1 (EqCO_2_-VT1)* (L)	32 (IQR 29–37)	31 (IQR 28.3–34.7)	0.005	31 (IQR 29–36)	30 (IQR 27–34)	0.235	34 (IQR 30–37.7)	32 (IQR 29–35)	0.002	+0.3	−1.6–2.2	0.778	1.000	0.001
*Peak VE/VO*_2_*(EqO*_2_*)* (L)	36 (IQR 30–43)	34 (IQR 29–40)	0.001	34 (IQR 30–42.5)	34 (IQR 28–40)	0.031	39 (IQR 32–43.5)	34 (IQR 31–41.5)	0.016	−0.1	−2.7–2.5	0.953	1.000	0.000
Gas exchange														
*Resting PETCO*_2_ (mmHg)	30.2 ± 4.8	29.8 ± 4.4	0.669	30.8 ± 4.9	30.0 ± 4.3	0.154	29.1 ± 4.5	29.5 ± 4.7	0.166	−1.0	−2.6–0.6	0.226	1.000	0.013
*PETCO*_2_*at VT1* (mmHg)	34.3 ± 5.5	38.8 ± 29.2	0.003	34.5 ± 5.8	40.9 ± 3.7	0.348	33.3 ± 4.8	35.5 ± 5.5	0.000	−0.8	−2.7–1.0	0.405	1.000	0.008
*Peak PETCO*_2_ (mmHg)	34.9 ± 6.2	36.4 ± 5.5	0.007	35.7 ± 6.2	36.6 ± 5.4	0.295	33.6 ± 6.1	36.1 ± 5.6	0.000	−0.8	−2.7–1.1	0.399	1.000	0.007
*Resting PETO*_2_ (mmHg)	115.7 ± 6.8	115.0 ± 6.0	0.243	115.0 ± 7.1	114.7 ± 5.6	0.926	116.8 ± 6.3	115.6 ± 6.7	0.095	0.0	−2.7–2.6	0.976	1.000	0.000
*Peak PETO*_2_ (mmHg)	118.5 ± 7.7	116.2 ± 7.1	0.000	117.2 ± 7.6	115.7 ± 7.0	0.015	120.7 ± 7.5	117.3 ± 7.1	0.002	+0.1	−2.1–2.2	0.908	1.000	0.000
Prognostic assessment														
*MECKI Score* (%)	4.4 (IQR 2.2–9.8)	1.2 (IQR 0.5–3.1)	0.000	3.9 (IQR 2.2–9.5)	0.6 (IQR 0.3–1.6)	0.000	5.9 (IQR 2.5–11.2)	3.3 (IQR 1.3–6.6)	0.000	−5.2	−12.0– (−1.7)	0.327	0.327	0.061

**Abbreviations: VO_2_ at VT1**: percentage of predicted oxygen uptake at the first ventilatory threshold; **HFimpEF**: heart failure with improved ejection fraction; **HFprEF**: heart failure with persistently reduced ejection fraction; **HFrEF**: heart failure with reduced ejection fraction; **HR at VT1:** heart rate at the first ventilatory threshold; **Max Exercise Time:** maximum exercise time; **MECKI Score:** Metabolic Exercise Combined Cardiac Kidney Index score; **O_2_Pulse**: oxygen pulse; **OUES**: oxygen uptake efficiency slope; **Peak HR**: peak heart rate; **Peak PETCO_2_**: peak end-tidal carbon dioxide partial pressure; **Peak PETO_2_**: peak end-tidal oxygen partial pressure; **Peak RER**: peak respiratory exchange ratio; **Peak VE/VO_2_ (EqO_2_)**: peak ventilatory equivalent for oxygen; **VO_2_Peak**: peak oxygen uptake; **PETCO_2_**: end-tidal carbon dioxide partial pressure; **PETCO_2_ at VT1**: end-tidal carbon dioxide partial pressure at the first ventilatory threshold; **PETO_2_**: end-tidal oxygen partial pressure; **Predicted OUES (%)**: predicted oxygen uptake efficiency slope; **Predicted VO_2_Peak (%)**: predicted peak oxygen uptake; **RER at 2 min recovery**: respiratory exchange ratio at 2 min recovery; **Resting HR**: resting heart rate; **Time at VT1**: time at the first ventilatory threshold; **VE/VCO_2_ at VT1**: ventilatory equivalent for carbon dioxide at the first ventilatory threshold; **VE/VCO_2_ slope**: minute ventilation to carbon dioxide output slope; **VO_2_ at VT1**: oxygen uptake at the first ventilatory threshold. (**†**) **ANCOVA (Bootstrap) model** compares the follow-up values between *HFimpEF* and *HFprEF* groups, using the baseline value of each variable as a covariate. Adjusted difference at follow-up, 95% Confidence Interval (95% CI), *p*-value, and Partial eta squared are based on 2000 bootstrap samples (BCa method).

**Table 4:** **Predictors of peak oxygen improvement. Univariate and Multivariate linear regression models**.

Univariate	B	95% CI	Beta	95% CI	*p*-Value	
LVEF Improvement	1.677	−0.020–0.357	0.168	−0.204–3.557	0.080	
Diabetes Mellitus	−1.957	−0.374–0.004	−0.184	−3.953–0.039	0.055	
Baseline PASP (mmHg)	−0.168	−0.531–0.004	−0.272	−3.338–0.003	0.054	
Baseline VO_2_Peak (mL/Kg/min)	−0.434	−0.595–(−0.251)	−0.425	−0.622–(−0.258)	<0.001	
Baseline %VO_2_Peak	−0.089	−0.520–(−0.163)	−0.343	−0.135–(−0.042)	<0.001	
VO_2_-VT1 (mL/Kg/min)	−0.379	−0.430–(−0.053)	−0.241	−0.675–(−0.083)	0.013	
%Predicted VO_2_-VT1	−0.096	−0.478–(−0.106)	−0.291	−0.156–(−0.035)	0.002	
Exercise Duration (min)	−0.197	−0.397–(−0.026)	−0.212	−0.369–(−0.024)	0.026	
Baseline O_2_ Pulse (mL/lat)	−0.363	−0.463–(−0.079)	−0.266	−0.620–(−0.106)	0.006	
Baseline % Predicted O_2_ Pulse	−0.072	−0.486–(−0.095)	−0.287	−0.120–(−0.024)	0.004	
Baseline QRS Duration (ms)	0.028	0.011–0.389	0.200	0.001–0.054	0.039	
**Multivariate**	**B**	**95% CI**	**Beta**	**95% CI**	**VIF**	***p*-value**
Age (years)	−0.118	−0.458 to −0.064	−0.261	−0.207 to −0.029	1.423	0.010
Female sex	−1.158	−0.458 to −0.064	−0.261	−3.183 to 0.868	1.385	0.259
Diabetes mellitus	−2.250	−0.306 to 0.083	−0.111	−4.190 to −0.310	1.202	0.023
Ischaemic etiology	0.581	−0.392 to −0.029	−0.210	−1.783 to 2.945	1.671	0.627
Heart failure duration (months)	0.010	−0.161 to 0.266	0.052	−0.012 to 0.032	1.432	0.369
Baseline VO_2_peak (mL/kg/min)	−0.580	−0.109 to 0.291	0.091	−0.774 to −0.387	1.319	<0.001
Baseline QRS duration (ms)	0.034	−0.759 to −0.379	−0.569	−0.012 to 0.079	4.021	0.142
Cardiac Rehabilitation Program	−1.164	−0.087 to 0.576	0.248	−3.210 to 0.882	1.577	0.261
CRT-LBBAP	−0.864	−0.326 to 0.089	−0.118	−4.374 to 2.646	4.848	0.626
ΔLVEF (percentage points)	0.019	−0.454 to 0.274	−0.090	−0.058 to 0.096	1.530	0.617

Model: n = 106 complete cases; R = 0.584; R^2^ = 0.341; adjusted R^2^ = 0.272; F(10,95) = 4.919, *p* < 0.001; Durbin–Watson = 1.532. **Abbreviations: B**: unstandardized coefficients; **Beta**: standardized coefficients; **CRT-LBBAP**, Cardiac Resynchronization Therapy via Left Bundle Branch Area Pacing; **LVEF**: Left Ventricular Ejection Fraction; **ΔLVEF:** LVEF at last follow-up minus baseline LVEF; **VIF:** variance inflation factor; **VO_2_peak**: peak oxygen uptake; **VO_2_ at VT1**: oxygen uptake at the first ventilatory threshold; **PASP**: Pulmonary Artery systolic Pressure.

## Data Availability

The data is available in its entirety upon request.

## Supplementary Materials

The following supporting information can be downloaded at https://www.mdpi.com/article/10.3390/jcm15218406/s1, Table S1. Comparative Analysis of Patient Cohorts, CPET parameters, and statistical methodology across the Agostoni et al. [12], Magrí et al. [15] and IMPRO_2_VEMENT studies.

## Abbreviations

**HFrEF:** Heart Failure with Reduced Ejection Fraction. **HFimpEF:** Heart Failure with Improved Ejection Fraction. **HFprEF:** Heart Failure with Persistently Reduced Ejection Fraction. **CPET**: Cardiopulmonary Exercise Test. **LVEDD***:* Left Ventricular End-Diastolic Diameter. **CRT-LBBAP**: Cardiac Resynchronization Therapy via Left Bundle Branch Area Pacing.

## References

[1] Mapelli M., Salvioni E., Mattavelli I., Vignati C., Galotta A., Magrì D., Apostolo A., Sciomer S., Campodonico J., Agostoni P. (2023). Cardiopulmonary exercise testing and heart failure: A tale born from oxygen uptake. Eur. Heart J. Suppl..

[2] Juarez M., Castillo-Rodriguez C., Soliman D., Del Rio-Pertuz G., Nugent K. (2024). Cardiopulmonary Exercise Testing in Heart Failure. J. Cardiovasc. Dev. Dis..

[3] Moneghetti K.J., Christle J.W., Myers J., Haddad F. (2018). Exercise testing in heart failure: A contemporary discussion in an era of novel diagnostic techniques and biomarkers. Curr. Opin. Cardiol..

[4] Malhotra R., Bakken K., D’elia E., Lewis G.D. (2016). Cardiopulmonary Exercise Testing in Heart Failure. JACC Heart Fail..

[5] Nadruz W., West E., Sengeløv M., Santos M., Groarke J.D., Forman D.E., Claggett B., Skali H., Shah A.M. (2017). Prognostic value of cardiopulmonary exercise testing in heart failure with reduced, midrange, and preserved ejection fraction. J. Am. Heart Assoc..

[6] Salvioni E., Bonomi A., Re F., Mapelli M., Mattavelli I., Vitale G., Sarullo F.M., Palermo P., Veglia F., Agostoni P. (2020). The MECKI score initiative: Development and state of the art. Eur. J. Prev. Cardiol..

[7] Lala A., Shah K.B., Lanfear D.E., Thibodeau J.T., Palardy M., Ambardekar A.V., McNamara D.M., Taddei-Peters W.C., Baldwin J.T., Jeffries N. (2021). Predictive Value of Cardiopulmonary Exercise Testing Parameters in Ambulatory Advanced Heart Failure. JACC Heart Fail..

[8] Swank A.M., Horton J., Fleg J.L., Fonarow G.C., Keteyian S., Goldberg L., Wolfel G., Handberg E.M., Bensimhon D., Illiou M.C. (2012). Modest increase in peak VO2 is related to better clinical outcomes in chronic heart failure patients: Results from Heart Failure and a Controlled Trial to Investigate Outcomes of Exercise Training. Circ. Heart Fail..

[9] Perea-Armijo J., López-Aguilera J., Sánchez-Prats R., Castillo-Domínguez J.C., González-Manzanares R., Ruiz-Ortiz M., Mesa-Rubio D., Anguita-Sánchez M., Prats R.S., Castillo-Dominguez J.C. (2023). Improvement of left ventricular ejection fraction in patients with heart failure with reduced ejection fraction: Predictors and clinical impact. Med. Clin..

[10] Bozkurt B., Coats A.J., Tsutsui H., Abdelhamid M., Adamopoulos S., Albert N., Anker S.D., Atherton J., Böhm M., Butler J. (2021). Universal Definition and Classification of Heart Failure: A Report of the Heart Failure Society of America, Heart Failure Association of the European Society of Cardiology, Japanese Heart Failure Society and Writing Committee of the Universal Definition of Heart Failure. J. Card. Fail..

[11] Brawner C.A., Lazar M.H. (2023). Cardiopulmonary exercise testing criteria for advanced therapies in patients with heart failure. Heart Fail. Rev..

[12] Agostoni P., Pluchinotta F.R., Salvioni E., Mapelli M., Galotta A., Bonomi A., Magrì D., Perna E., Paolillo S., Corra U. (2023). Heart failure patients with improved ejection fraction: Insights from the MECKI score database. Eur. J. Heart Fail..

[13] Hansen D., Abreu A., Ambrosetti M., Cornelissen V., Gevaert A., Kemps H., Laukkanen J.A., Pedretti R., Simonenko M., Wilhelm M. (2022). Exercise intensity assessment and prescription in cardiovascular rehabilitation and beyond: Why and how: A position statement from the Secondary Prevention and Rehabilitation Section of the European Association of Preventive Cardiology. Eur. J. Prev. Cardiol..

[14] Gitt A.K., Wasserman K., Kilkowski C., Kleemann T., Kilkowski A., Bangert M., Schneider S., Schwarz A., Senges J. (2002). Exercise anaerobic threshold and ventilatory efficiency identify heart failure patients for high risk of early death. Circulation.

[15] Magrì D., Piepoli M., Corrà U., Gallo G., Maruotti A., Vignati C., Salvioni E., Mapelli M., Paolillo S., Filardi P.P. (2020). Cardiovascular Death Risk in Recovered Mid-Range Ejection Fraction Heart Failure: Insights From Cardiopulmonary Exercise Test. J. Card. Fail..

[16] Sato T., Yoshihisa A., Kanno Y., Suzuki S., Yamaki T., Sugimoto K., Kunii H., Nakazato K., Suzuki H., Saitoh S.I. (2017). Cardiopulmonary exercise testing as prognostic indicators: Comparisons among heart failure patients with reduced, mid-range and preserved ejection fraction. Eur. J. Prev. Cardiol..

[17] Corrà U., Giordano A., Gambarin F.I., Gnemmi M., Marcassa C., Pistono M. (2022). Cardiopulmonary exercise testing in patients with asymptomatic left ventricular dysfunction: Lack of prognostic predictive power of ventilatory variables. Eur. J. Prev. Cardiol..

[18] Tokavanich N., Prasitlumkum N., Mongkonsritragoon W., Trongtorsak A., Cheungpasitporn W., Chokesuwattanaskul R. (2022). QRS area as a predictor of cardiac resynchronization therapy response: A systematic review and meta-analysis. PACE—Pacing Clin. Electrophysiol..

[19] Gutiérrez-Ballesteros G., Mazuelos-Bellido F., López-Aguilera J., Crespín-Crespín M., González-Manzanares R., García-Merino M.A., Mesa-Rubio D., Romero-Moreno M., Pan Álvarez-Osorio M., Segura Saint-Gerons J.M. (2025). Changes in oxygen uptake in patients with non-ischemic dilated cardiomyopathy and left bundle branch block following left bundle branch area pacing. Front. Cardiovasc. Med..

[20] Solano J., Rahunathan N., Sykes D.L., Eni G., Edhem L., Witte K.K. (2025). The effect of Cardiac Resynchronization Therapy (CRT) on functional capacity based on Cardiopulmonary Exercise Testing (CPET): A systematic review and meta-analysis. Eur. Heart J. Open.

[21] Pezzuto B., Piepoli M., Galotta A., Sciomer S., Zaffalon D., Filomena D., Vignati C., Contini M., Alimento M., Baracchini N. (2023). The importance of re-evaluating the risk score in heart failure patients: An analysis from the Metabolic Exercise Cardiac Kidney Indexes (MECKI) score database. Int. J. Cardiol..

