# Supplementary material for: Longitudinal Assessment of Exercise Capacity via Cardiopulmonary Exercise Testing in Heart Failure Patients with Improved and Persistently Reduced Ejection Fraction: Results from the IMPRO2VEMENT-HF Study
Source: J Clin Med. 2026 Oct 28;15(21):8406. doi: 10.3390/jcm15218406 (PMC13763660; doi:10.3390/jcm15218406)
Supplement: Supplementary file 1 [file jcm-15-08406-s001.zip › jcm-4467863-supplementary.pdf]

Table S1. Comparative Analysis of Patient Cohorts, CPET parameters, and statistical methodology across the Agostoni et al [12], Magrí et al [15] and *IMPRO2VEMENT* studies.

Feature of Study	Agostoni et al (2023) [12]	Magrí et al (2020) [15]	*IMPRO2VEMENT* Study
**Study Design**	Multicenter, prospective/retrospective registry (MECKI score database)	Multicenter, prospective/retrospective registry (MECKI score database)	Single-center, longitudinal retrospective cohort
**Analyzed Population**	**N = 7521** (1504 *HFimpEF* vs. 6017 *HFprEF*)	**N = 5711** (1176 *rec-HFmrEF* vs. 4535 *HFrEF*)	**N = 111** (69 *HFimpEF* vs. 42 *HFprEF*)
**Temporal Assessment**	Cross-sectional / Data Analyzed at a single time point	Cross-sectional / Data Analyzed at a single time point	Longitudinal (Baseline vs. Follow-up comparison at approximately 10 months)
**Between-Group Change Analysis**	Mann-Whitney U / Chi-square tests (Cross-sectional comparison)	Mann-Whitney U / Chi-square tests (Cross-sectional comparison)	ANCOVA with Bootstrap (Adjusted for baseline differences between groups)
**Absolute *VO2peak* (mL/kg/min)**	*HFimpEF*: 15.5 [12.2 – 19.3]<br>*HFprEF*: 14.0 [11.3 – 17.2], p<0.001	*rec-HFmrEF*: 16.1 ± 5.5<br>*HFrEF*: 14.4 ± 4.5, p<0.001	*HFimpEF*: 15.5 -> 19.2 (p<0.001)<br>*HFprEF*: 15.7 -> 17.7 (p<0.001)<br>*ANCOVA adjusted difference* 1.6; p=0.072
**%Predicted *VO2peak***	*HFimpEF*: 62.2% [50.7 – 74.1]<br>*HFprEF*: 52.6% [41.8 – 64.3], p<0.001	*rec-HFmrEF*: 63 ± 18%<br>*HFrEF*: 53 ± 16%, p<0.001	*HFimpEF*: 63% -> 78% (p<0.001)<br>*HFprEF*: 60% -> 68% (p=0.167)<br>*ANCOVA adjusted difference* +8.0; p=0.021
**VE/VCO2 slope**	*HFimpEF*: 30.0 [26.9 – 34.4]<br>*HFprEF*: 32.1 [28.0 – 38.0], p<0.001	*rec-HFmrEF*: 30.8 ± 6.5<br>*HFrEF*: 33.4 ± 8.5, p<0.001	*HFimpEF*: 29.0 -> 27.0 (p=0.025)<br>*HFprEF*: 33.0 -> 29.6 (p<0.001)<br>*ANCOVA adjusted difference* -0.4; p=0.755
**MECKI Score (%)**	*HFimpEF*: 1.84% [0.99 – 3.27]<br>*HFprEF*: 7.18% [3.41 – 14.7], p<0.001	Not evaluated as a comparative baseline variable	*HFimpEF*: 3.9% -> 0.6% (p<0.001)<br>*HFprEF*: 5.9% -> 3.3% (p<0.001)<br>*ANCOVA adjusted difference* -5.2; p=0.327
**Recovery Delay (msec)**	Not analyzed	Not analyzed	*HFimpEF*: 30 -> 29 (p=0.057)<br>*HFprEF*: 35-> 30 (p=0.601)<br>*ANCOVA adjusted difference* -9.0 msec; p=0.047

Abbreviations: ANCOVA: Analysis of Covariance; **CPET**: cardiopulmonary exercise test; **HFimpEF**: Heart Failure with Improved Ejection Fraction; **HFprEF**: Heart Failure with Persistently Reduced Ejection Fraction; **MECKI Score**: Metabolic Exercise Combined Cardiac Kidney Index score; **rec-HFmrEF**: Heart Failure with Recovered Mid-range Ejection Fraction; **VE/VCO2 slope**: minute ventilation to carbon dioxide output slope; **VO2peak**: Peak Oxygen Uptake.
